# Prevalence, socio-demographics and service use determinants associated with disclosure of HIV/AIDS status to infected children: a systematic review and meta-analysis by 1985–2021

**DOI:** 10.1186/s13690-022-00910-6

**Published:** 2022-06-09

**Authors:** Bahram Armoon, Marie-Josée Fleury, Peter Higgs, Amir-Hossien Bayat, Azadeh Bayani, Rasool Mohammadi, Elaheh Ahounbar

**Affiliations:** 1 Douglas Mental Health University Institute, Research Centre, 6875 LaSalle Boulevard, Montreal, QC H4H 1R3 Canada; 2 Department of Psychiatry, McGill University, Montreal, QC Canada; 3 Department of Public Health, La Trobe University, Melbourne, Australia; 4 Burnet Institute, Melbourne, VIC Australia; 5 Social Determinants of Health Research Center, Saveh University of Medical Sciences, Saveh, Iran; 6 Student Research Committee, School of Allied Medical Sciences, Shahid Beheshti University of Medical Sciences, Tehran, Iran; 7 Department of Biostatistics and Epidemiology, School of Public Health and Nutrition, Lorestan University of Medical Sciences, Khorramabad, Iran; 8 Orygen, The National Center of Excellence in Youth Mental Health, University of Melbourne, Parkville, VIC Australia; 9 Center for Youth Mental Health, Faculty of Medicine, Dentistry and Health Sciences, University of Melbourne, Parkville, Australia

**Keywords:** Disclosure of HIV/AIDS, antiretroviral treatment, hospital follow-up, social support

## Abstract

**Background:**

Human immunodeficiency virus (HIV)/Acquired Immune Deficiency Syndrome (AIDS) is a public health issue of global importance. To our knowledge, no previous meta-analysis documenting the prevalence, socio-demographic, and service use determinants associated with HIV/AIDS disclosure to infected children has been conducted. The present study aimed to determine the prevalence, socio-demographics and service use determinants associated with the disclosure of HIV/AIDS status to infected children.

**Methods:**

Studies in English published between 01 January 1985 and 01 November 2021, and available on PubMed, Scopus, Web of Science, and Cochrane electronic databases were searched. After reviewing for study duplicates, the full-text of selected articles were assessed for eligibility using Population, Intervention, Comparator, Outcomes (PICO) criteria. We used fixed and random-effects meta-analysis models to estimate the pooled prevalence, pooled odds ratio (OR), and 95% confidence intervals.

**Results:**

After article duplicates were excluded, assessments of abstracts were completed, and full-text papers evaluated, 37 studies were included in this meta-analysis. The prevalence of the disclosure of HIV status to children was measured to be 41% in this research. The odds that a child of 10 years and older is informed that they are HIV-positive is 3.01 time the odds that younger children are informed. Those children who had primary or lower schooling level were 2.41 times more likely to be informed of their HIV-positive status than children with higher levels of schooling. Children who had a non-biological parents were 3.17 times more likely to have been disclose being HIV-positive; social support (OR = 8.29, 95%CI = 2.34, 29.42), children who had higher levels of social supports were 8.29 times more likely to disclose HIV-positive; the primary educational level of caregivers (OR = 2.03, 95%CI = 1.43, 2.89), respondents who had caregivers with primary education level were 2.03 times more likely to disclose HIV-positive; antiretroviral treatment (ART) adherence (OR = 2.59, 95%CI = 1.96, 3.42), participants who adhered to ART were 2.59 times more likely to disclose HIV-positive and hospital follow-up (OR = 2.82, 95%CI = 1.85, 4.29), those who had hospital follow-up were 2.82 times more likely to disclose HIV-positive; were all significantly associated with the disclosure of HIV/AIDS status to infected children.

**Conclusion:**

Such data are of importance for healthcare pediatrics HIV care professionals. Facilitating HIV diagnosis and disclosure to the infected children and ensuring access to HIV treatment will likely prevent secondary HIV transmission. Healthcare professionals are expected to provide age-appropriate counseling services to this population.

**Supplementary Information:**

The online version contains supplementary material available at 10.1186/s13690-022-00910-6.

## Background

Human immunodeficiency virus (HIV)/Acquired immune deficiency syndrome (AIDS) has a negative impact on the lives of both children and adults, and disclosure is a psychological challenge for health care providers and families deciding how and when to disclose HIV positive status to the affected patients [1]. The disclosure of HIV status is a complex social issue and producing positive and/or negative impacts upon individuals. One positive side of HIV disclosure is that it can increase quality of life because the HIV positive patients receive the financial, psychological, and even physical support from family members and others [2]. HIV positive patients who receive social support have been found to have more self-esteem, better adaptation, and a better lifestyle [3]. Research suggests that they less anxiety related to hiding their HIV status, and stress and risk behaviors have been found to decrease [4].

Alternatively non-disclosure of HIV has detrimental effects on children with those who are unaware of their HIV-positive status, less likely to follow their medication process regularly, leading to death and drug resistance [5]. Disclosure has a number of positive clinical outcomes including adherence and viral suppression though mental health problems in individuals who have lived with HIV for a long time have also been reported [5].

There are several numbers of factors that impact HIV disclosure. HIV-related stigma has been shown to make disclosure difficult, and evidence suggests that caregivers postpone disclosure because of the fear of subsequent discrimination and stigma for themselves and their children [6]. Social support is a necessary resource for coming to terms with living with HIV, and it is probable psychological problems such as depression are reduced when this support is available [7].

Improving global access to antiretroviral treatment (ART) for children not only reduces HIV-related child mortality but increases the number of people living with HIV [8]. HIV disclosure encourages safer sex behaviors in younger people and boosts access to social support. Disclosure of HIV status is beneficial as children approach adolescence and transition to adulthood. Disclosure has been found to improve adherence to medication and increasing awareness of HIV enables effective participation in patient treatment and self-care [9, 10]. Knowledge of HIV status while moving into adolescence is vital as it provides opportunities for self-responsibility and management of their treatment [11]. Despite the merits of the disclosure there is no universally agreed time or age when HIV positive status should be communicated.

While previous systematic reviews related to HIV/AIDS disclosure in children and adults have been conducted [1, 12–19] they have been narrow in focus. Previous systematic reviews have investigated the psychosocial determinants associated with disclosure of HIV/AIDS status among children and adults [1, 12, 13, 15–17, 19] or have looked at the effect of disclosure on adherence to antiretroviral therapy [18] or the perception of caregivers to the disclosure of HIV/AIDS status [14]. However, no previous study has conducted a meta-analysis concerning the prevalence, socio-demographics, and service use determinants associated with disclosure of HIV/AIDS status to infected children globally. Given this, a better understanding of the outcomes of disclosing HIV/AIDS status to infected children on socio-demographics and the determinants of service use may improve HIV treatment management strategies, including adherence to treatment for children. The present systematic review and meta-analysis study aimed to determine the prevalence, socio-demographics and service use determinants associated with the disclosure of HIV/AIDS status to infected children.

## Methods

### Search strategy

Our study was implemented using the Protocols of Systematic Reviews and Meta-Analyses (PRISMA) guidelines [20–25]. According to the search strategy and additional manual searches from the article references, 11,261 articles from four databases were found and screened. For the article inclusion, two independent researchers (AB and BA) reviewed the electronic databases of PubMed, Scopus, Web of Science, and Cochrane electronic database from January 1st, 1985 to November 1st, 2021. All fields within records and Medical Subject Headings (MeSH terms) were used to expand the search in these databases. The search strategy was prepared and modified for the various databases using important Boolean operators (AND/OR) with initial keywords “(Social Determinants of Health), (Socioeconomic Factors), (Spouses), (Literacy), (Medication Adherence), (CD4 Lymphocyte Count), (Health Services Accessibility), (Time-to-Treatment), (Time to Diagnosis), (Time to diagnosis Title/Abstract), (previous testing Title/Abstract), (Previous Testing Title/Abstract), (Social Stigma), (Stigma) (Self-Disclosure), (Self-Concept), (Anti-HIV Agents), (Antiretroviral Therapy), (Social Support), (People Who Lived with HIV Title/Abstract), (Living with HIV Title/Abstract), (HIV), (Child Day Care Centers), (Child), (Adult Children), (Child, Preschool)”(Supplementary File 1). The references were managed by EndNote X7 software (Thomson Reuters). Duplicate articles were excluded.

### Inclusion criteria based on based on population, intervention, comparison, and outcome (PICO)

We reviewed cross-sectional, cohort, and case-control studies. According to the PICO criteria, for the “population,” only children below who is below the age of 18 years living with HIV were included; the “intervention” targeted children who disclosures HIV status; the “comparison” group was children not reporting HIV status disclosure; “outcomes” were the significant association of the social-demographic factors and service use determinant of HIV infection disclosure status to infected children.; “study design” included cross-sectional, cohort, or case-control studies. Qualitative studies, secondary studies not reporting primary data, systematic reviews, and meta-analysis studies were excluded. Articles with significant heterogeneity or outcome variations from the study groups were excluded. Articles or variables that were not investigated extensively enough to be included in the meta-analysis were also not considered as associated variables of HIV infection disclosure status to infected children (i.e., being female, quality of life, child age (> 10 years) when ART started, family number > 3, death of a family member, World Health Organization (WHO) stage of HIV).

### Study selection and data extraction

Initially, two researchers reviewed the extracted article titles and abstracts independently, based on PICO criteria. A third member of the research team (AMB) provided extra input and resolved disagreements about articles to be included in the study. Secondly, AB and BA assessed the full articles, considering the study inclusion criteria based on PICO and exclusion criteria, including having no access to the full article and manuscripts missing principal data. Only articles written in English were included. Two researchers (AB and BA) evaluated the studies separately, applying a standardized data collection (excel spreadsheet) form. Any contradictions or differences in opinions about the quality of the overall studies between the authors were resolved by senior author EA and first author AB through discussion. The surname of the first author, publication year, prevalence, socio-demographic data of participants (age less than 10 years, education, non-biological parents), and other features such as service use determinants such as medication adherence, hospital follow-up, time since diagnosis, and accessibility to care were recorded during the data extraction.

### Study quality assessment

The Newcastle-Ottawa Scale (NOS) [26] recommended by the Cochrane Collaboration [27] was implemented to examine the quality of the reviewed studies (Supplementary File 2) in terms of exposure, outcome, and comparability with a scale of very good, good, satisfactory, and unsatisfactory quality domains. This scale consisted of three domains of selection, comparability, and exposure/outcome which each of them included 3, 1, and 1 item for cross-section studies. The agreement levels of poor, slight, fair, moderate, substantial, and almost perfect were considered by the values 0, 01–0.02, 0.021–0.04, 0.041–0.06, 0.061–0.08, and 0.081–1.00, respectively [28].

### Data synthesis and statistical analysis

The meta-analysis was produced by pooling odds ratios (OR) with 95% confidence intervals recognizing socio-demographic and service use determinants associated with HIV infection disclosure to children. We applied the Q test with a *P* value < 0.05 and I^2^ statistics with a cutoff of ≥50% to evaluate the correlations across the studies. We also obtained uncertainty 95% confidence intervals for I^2^. We assumed any negative values to I^2^ as equal to zero. We used the random-effects model to compute pooled estimations, taking into account the different sampling methods of the selected studies. To recognize any publication bias, Egger’s approach was performed both graphically and statistically [29, 30]. We considered the *P*-value of 0.05 as statistically significant. The association between socio-demographics and service use determinants associated with HIV infection disclosure status to infected children were proposed by an OR and 95% CI. We visualized the obtained results in forest plots. For data analysis, R 3.5.1 with the “meta” package was applied to perform the meta-analysis.

## Results

### Study characteristics

**Fig. 1:**
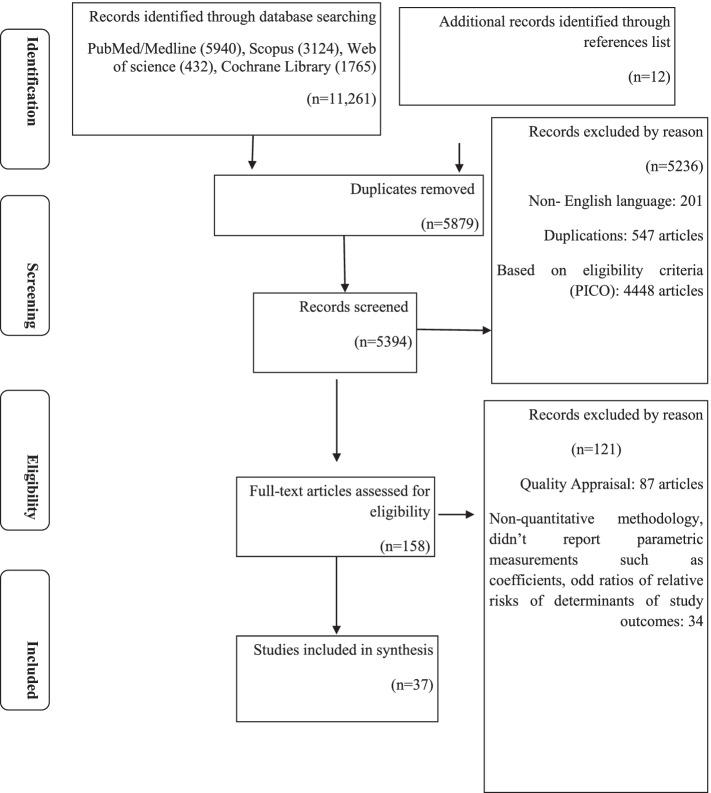
PRISMA flow diagram, systematic review and meta-analysis on the disclosure of HIV/AIDS status to infected children, 1985–2021

The study selection process is shown in Fig. 1. There were 11,261 published papers found in the four databases searched and including the references from the papers reviewed. After article duplicates were excluded, assessments of abstracts completed, and full-text papers evaluated, 37 studies were retained for inclusion in this meta-analysis [5, 9, 31–65]. Of the 37 studies, 34 were based on data collected from the African Region (*n* = 22,351 participants) and three from the South-East Asian Region (*n* = 549 participants). Ethiopia was the country with the highest number of included studies (12 studies and 3669 participants). Considering the World Bank country income level, there were 7 studies (*n* = 3042) from an upper middle income country included, 15 studies (*n* = 14,240) were from lower middle income countries and 15 studies (*n* = 4584) were from lower-income countries. No studies from either high or middle-income countries were included.

### Results of the meta-analysis

We analyzed the association of the following socio-demographics: child aged less than 10 years old, having primary or lower school education level, having non-biological parents, primary education of caregivers and having social support), and service use determinants (duration of ART, ART therapy adherence, hospital follow up and time since diagnosis) associated with disclosure of HIV/AIDS status to infected children (Table 1).

**Table 1:** Main characteristics of the studies included in the systematic review and meta-analysis on the disclosure of HIV/AIDS status to infected children, 1985–2021

Author	Sample size	Year of publish	Year of implementation	Design	Quality of the evidence	Socio-demographic determinants					Service use determinants			
						Child aged higher than 10 years	Primary or lower schooling level	Having a non-biological parents	Higher social support	Primary education of caregivers	Medication adherence (ART therapy)	Hospital follow-up	Time since diagnosis of 5 years or more	Duration of ART more than 5 years
Appiah et al. [1]	180	2021	2017–2018	Cross-section	Good						*			
Abegaz et al. [2]	449	2019	2018	Cross-section	Satisfactory	*				*		*	*	
Alemu et al. [3]	231	2013	2013	Cross-section	Good								*	
Lencha et al. [4]	200	2018	2018	Cross-section	Good				*					
Negese et al. [5]	428	2012	2012	Cross-section	Good			*						
Meena et al. [6]	144	2018	2015–2017	Cross-section	Satisfactory									
Bajaria et al. [7]	10,673	2020	2018–2019	Cross-section	Satisfactory		*			*				
Bulali et al. [8]	39	2018	2017	Cross-section	Good		*				*			
Namasopo-Oleja et al. [9]	174	2015	2009	Cross-section	Very Good	*			*		*			
Atwiine et al. [10]	312	2015	2012	Cross-section	Good									
Mengesha et al. [11]	325	2018	2016	Cross-section	Good									
Vreeman et al. [12]	285	2015	2013	Cross-section	Very Good									
Paintsil et al. [13]	446	2020	2013–2016	Cross-section	Good					*				
Cluver et al. [14]	1102	2015	2015	Cross-section	Good									
Nzota et al. [15]	334	2015	2011–2012	Cross-section	Good	*								
Ayele et al. [16]	374	2021	2019	Cross-section	Very Good	*			*					
Tamir et al. [17]	300	2015	2005	Cross-section	Very Good						*			*
Bhattacharya et al. [18]	145	2011	2010	Cross-section	Very Good		*	*		*	*		*	
Okechukwu et al. [19]	218	2017	2018	Cross-section	Good	*								
Guta et al. [20]	221	2020	2019	Cross-section	Satisfactory	*								*
van Elsland et al. [21]	190	2019	2012–2013	Cross-section	Good	*								
Madiba et al. [22]	218	2013	2009–2010	Cross-section	Good	*		*						
Shallo and Tassew [23].	247	2020	2019	Cross-section	Very Good									
Finnegan et al. [24]	372	2019	2019	Cross-section	Very Good		*							
Kallem et al. [25]	71	2011	2009	Cross-section	Good	*							*	*
Madiba and Mokgatle [26].	405	2013	2017	Cross-section	Good					*				
Danjuma et al. [27]	160	2021	2017	Cross-section	Satisfactory	*								
John-Stewart et al. [28]	271	2013	2006–2007	Cross-section	Good									
Murnane et al. [29]	553	2017	2013–2014	Cross-section	Good									
Odiachi and Abegunde [30].	110	2015	2015	Cross-section	Good	*								
Beima-Sofie et al. [31]	314	2017	2013	Cross-section	Very Good									
Tucho et al. [32]	327	2021	2020	Cross-section	Good	*						*		*
Biadgilign et al. [33]	390	2008	2011	Cross-section	Very Good	*						*		
Vreeman et al. [34]	792	2014	2011–2012	Cross-section	Very Good	*					*			
Tadesse et al. [35]	177	2015	2015	Cross-section	Good	*					*		*	
Kalembo et al. [36]	429	2019	2015	Cross-section	Good	*								
Sirikum et al. [37]	260	2014	2014	Cross-section	Good	*								

*: Variables that are included in the meta-analysis

#### Prevalence of disclosure of HIV/AIDS status to infected children

**Fig. 2:**
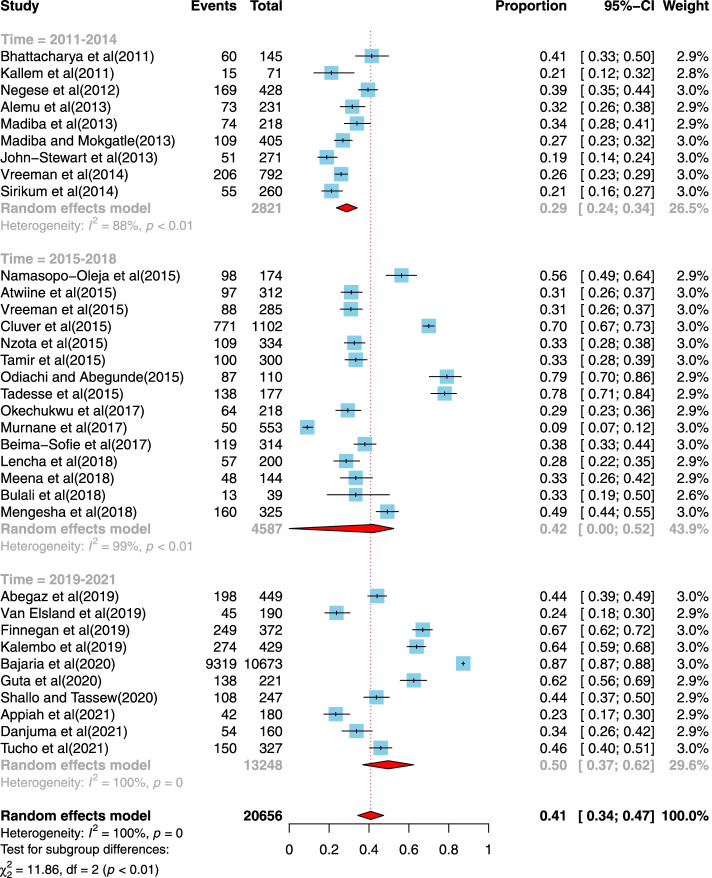
Forest plot displaying the pooled prevalence of disclosure of HIV/AIDS status to infected children

Thirty-four studies [5, 9, 31–41, 43, 44, 46–59, 61–64] reported the prevalence rate of disclosure of HIV/AIDS status to infected children. Thirteen studies were from a low income country setting [5, 32–34, 38–40, 46, 49, 51, 59, 62, 63], fourteen studies were in a lower middle income country setting [31, 35–37, 41, 44, 47, 48, 52, 53, 55, 56, 58, 61] and eight were from a upper-middle-income country [9, 43, 50, 54, 57, 64, 65]. The studies were published between 2008 and 2021, and the sample sizes ranged from 39 to 10,673. Most studies were conducted in Ethiopia (*n* = 12), and South Africa was second-ranked (*n* = 5). Twenty-one studies were assessed as good quality study designs. Figure 2 shows the pooled prevalence rate of disclosure of HIV/AIDS status to infected children to be 41% (95% CI, 34, 47%). We ran a subgroup analysis based on time of publication of studies and categorized the studies into three time slots: a) 2011–2014, b) 2015–208 and c) 2019–2021. We found that HIV disclosure has improved over time from 2011 to 2014 where it was 29% (95% CI, 24, 34%) then in 2015–2018 42% (95% CI, 0, 52%) and finally in 2019–2021 it was 50% (95% CI, 37, 62%).

#### Socio-demographic determinants associated with disclosure of HIV/AIDS status

##### The association between children aged higher than 10 years and disclosure of HIV/AIDS status to infected children

**Fig. 3:**
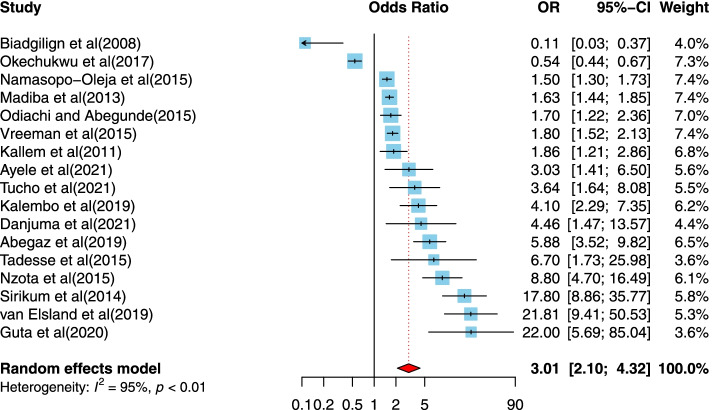
Forest plots for pooled odds ratio of the association between child aged higher than 10 years compared to lower than 10 years and disclosure of HIV/AIDS status to infected children (OR larger than 1 indicates that the odds of being inform on the HIV/AIDS status is higher in children aged 10 years and above compared to younger children)

Seventeen of the studies [5, 38, 41, 44, 45, 48–50, 53, 55, 58–60, 62–65] examined the association of being aged older than 10 years and disclosure of HIV/AIDS status. Three studies were from a upper-middle-income country [50, 64, 65], six studies were conducted in a lower middle income country setting [41, 44, 48, 53, 55, 58] and eight of the published studies used data from a low income country seating [5, 38, 45, 49, 59, 60, 62, 63]. The studies were published between 2008 and 2021, and the sample sizes ranged from 174 to 449. All studies used a cross-sectional design. Most studies were conducted in Ethiopia (*n* = 6) and 10 studies were evaluated as good quality designs. The positive association between older than 10 years compared to being aged lower than 10 years the HIV/AIDS diagnosis was provided to infected children is shown in Fig. 3**.** The source of heterogeneity that has been achieved is 95.0%. (OR = 3.01, 95%CI = 2.1, 4.32).

##### The association between primary or lower schooling level and disclosure of HIV/AIDS status to infected children

**Fig. 4:**
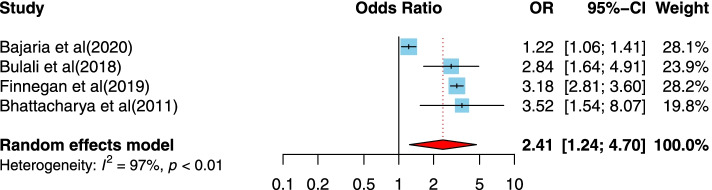
Forest plots for pooled odds ratio of the association between primary or lower schooling level compared to higher schooling level and disclosure of HIV/AIDS status to infected children (OR larger than 1 indicates that the odds of being inform on the HIV/AIDS status is higher in children who had primary or lower schooling level compared to higher schooling level)

Four studies [36, 37, 47, 52] examined the association of primary or lower schooling levels and disclosure of HIV/AIDS status to infected children. All studies were from a lower-middle-income country. The studies were published between 2011 and 2020, and the sample sizes ranged from 39 to 10,673. All studies used a cross-sectional design. Two studies were conducted in Tanzania, and two studies were assessed as high-quality designs. Our finding indicates a positive association between primary or lower schooling level and disclosure of HIV/AIDS status to infected children. Children who had primary or lower schooling levels were more likely to have reported disclosure of HIV/AIDS compared to children who had higher schooling levels (OR = 2.41, 95%CI = 1.24, 4.7), and the heterogeneity is about 97%, indicating variability in the data (Fig. 4).

##### The association between having non-biological parents and disclosure of HIV/AIDS status to infected children

**Fig. 5:**
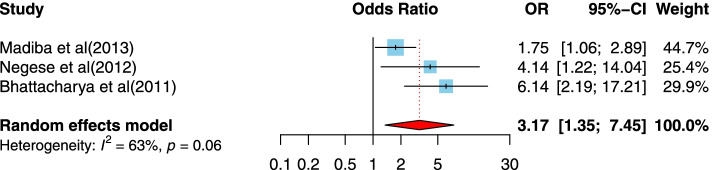
Forest plots for pooled odds ratio of the association between having a non-biological parents compared to biological parents and disclosure of HIV/AIDS status to infected children (OR larger than 1 indicates that the odds of being inform on the HIV/AIDS status is higher in children who had a non-biological parents compared to biological parents)

Three of the included studies [34, 47, 50] examined the association between having a non-biological parents and disclosure of HIV/AIDS status to infected children. One study was from an upper-middle-income country setting [50], one study was conducted in a lower-middle-income country setting [47], and one study was in a low-income country setting [34]. The studies were published between 2011 and 2013, and the sample sizes ranged from 145 to 428. All studies used a cross-sectional design. One of the studies was conducted in India, one of them was in Ethiopia, and the other one in South Africa. Two studies were assessed as having suitable quality designs. The positive association between having non-biological parents compared to biological parents and disclosure of HIV/AIDS status to infected children is shown in Fig. 5, and the source of heterogeneity that has been achieved is 63.0%. (OR = 3.17, 95%CI = 1.35, 7.45).

##### The association between high social support and disclosure of HIV/AIDS status to infected children

**Fig. 6:**
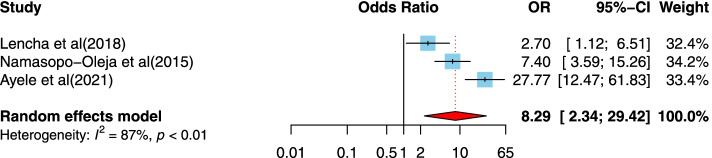
Forest plots for pooled odds ratio of the association between higher social support compared to lower social support and disclosure of HIV/AIDS status to infected children (OR larger than 1 indicates that the odds of being inform on the HIV/AIDS status is higher in children who had higher social support compared to lower social support)

Three studies [33, 38, 45] examined the association between social support and disclosure of HIV/AIDS status to infected children. All studies were from lower-income countries. The studies were published between 2015 and 2021, and the sample sizes ranged from 174 to 374. All studies used a cross-sectional design. Two studies were conducted in Ethiopia, and two studies were assessed as high-quality designs. Our finding indicates a positive association between social support and disclosure of HIV/AIDS status to infected children. Children who had social supports were more likely to have been provided with their HIV diagnosis compared to those who had no social supports (OR = 8.29, 95%CI = 2.34, 29.42), and the heterogeneity is about 87%, indicating variability in the data (Fig. 6).

##### The association between the primary education levels of caregivers and disclosure of HIV/AIDS status to infected children

**Fig. 7:**
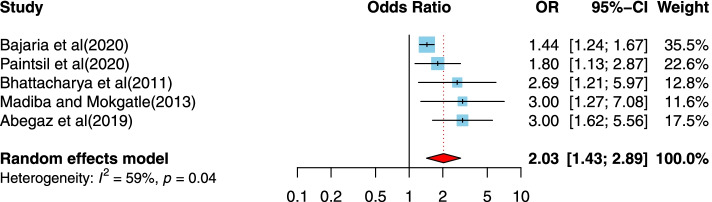
Forest plots for pooled odds ratio of the association between primary education of caregivers compared to high education and disclosure of HIV/AIDS status to infected children (OR larger than 1 indicates that the odds of being inform on the HIV/AIDS status is higher in children who had a caregiver with primary education compared to those with high education)

Five of the studies [5, 36, 42, 47, 54] examined the association between the primary education levels of caregivers and the disclosure of HIV/AIDS status to infected children. One study was from an upper-middle-income country setting [54], three studies were conducted in a lower-middle-income country setting [36, 42, 47], and one study was in a low-income country setting [5]. The studies were published between 2011 and 2020, and the sample sizes ranged from 145 to 10,673. All studies used a cross-sectional design. For five studies, one was conducted in Ethiopia, one in Ghana, one in Tanzania, one in India, and one in South Africa. Two of them were assessed as having suitable quality study designs. Our finding indicates a positive association between the primary education of caregivers and disclosure of HIV/AIDS status to infected children. Compared to children whose caregivers had higher levels of education, children who had caregivers with a primary level education were more likely to have reported disclosure of HIV/AIDS compared to those who had higher education levels (OR = 2.03, 95%CI = 1.43, 2.89). The heterogeneity is about 59%, indicating variability in the data (Fig. 7).

#### Service use determinants associated with disclosure of HIV/AIDS status

##### The association between medication adherence (ART therapy) and disclosure of HIV/AIDS status to infected children

**Fig. 8:**
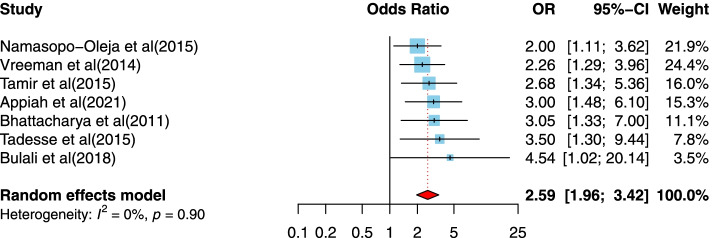
Forest plots for pooled odds ratio of the association between medication adherence (ART therapy) compared to no medication adherence and disclosure of HIV/AIDS status to infected children (OR larger than 1 indicates that the odds of being inform on the HIV/AIDS status is higher in children who had medication adherence (ART therapy) compared to no medication adherence)

Seven cross-sectional studies [31, 37, 38, 46, 47, 61, 62] examined the relationship between ART adherence and disclosure of HIV/AIDS status to infected children. Four studies were conducted in a lower-middle-income country setting [31, 37, 47, 61], and three studies were conducted in a low-income country setting [38, 46, 62]. Studies were published between 2011 and 2021, with sample sizes ranging from 39 to 792. Two studies were conducted in Ethiopia, and four studies were assessed as high-quality designs. As illustrated in Fig. 8, medication adherence has a positive association with the reporting of disclosure of HIV/AIDS status to infected children. The heterogeneity statistic is about 0%, and the pooled effect size implies a relatively neutral association. Compared to participants reporting no medication adherence, those who reported medication adherence were 2.59 times more likely to report having been informed about their HIV/AIDS status (OR = 2.59, 95%CI = 1.96, 3.42) (Fig. 8).

##### The association between hospital follow-up and disclosure of HIV/AIDS status to infected children

**Fig. 9:**
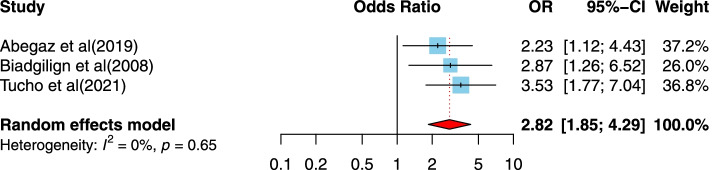
Forest plots for pooled odds ratio of the association between hospital follow-up compared to non-hospital follow-up and disclosure of HIV/AIDS status to infected children (OR larger than 1 indicates that the odds of being inform on the HIV/AIDS status is higher in children who had hospital follow-up compared to non-hospital follow-up)

Three studies [5, 59, 60] examined the association between hospital follow-up and disclosure of HIV/AIDS status to infected children; all studies were from low-income countries. The studies were published between 2008 to 2021, and the sample sizes ranged from 327 to 449. All three studies used a cross-sectional design. All studies were conducted in Ethiopia, and one of the studies had high-quality structures. The hospital follow-up results are presented in Fig. 9 and show a positive association between hospital follow-up and disclosure of HIV/AIDS status to infected children. Compared to those who reported non-hospital follow-up, those who reported follow-up after hospitalization were more likely to report disclosure of HIV/AIDS (OR = 2.82, 95%CI = 1.85, 4.29), and the heterogeneity is 0% (Fig. 9).

##### The association between the duration of ART of more than 5 years and disclosure of HIV/AIDS status to infected children

**Fig. 10:**
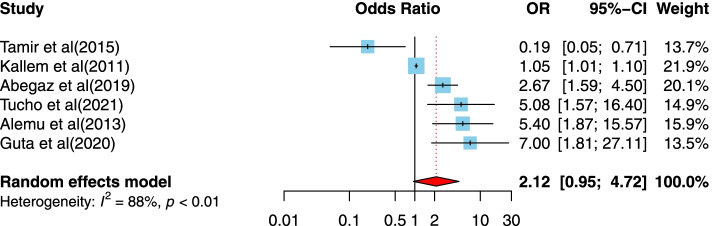
Forest plots for pooled odds ratio of the association between duration of ART more than 5 years compared to ≤5 years and disclosure of HIV/AIDS status to infected children (OR larger than 1 indicates that the odds of being inform on the HIV/AIDS status is higher in children who had duration of ART more than 5 years compared to ≤5 years)

Six cross-sectional studies [5, 32, 46, 49, 53, 59] examined the relationship between the duration of ART of more than 5 years compared to ≤5 years and disclosure of HIV/AIDS status to infected children. Five studies were conducted in a low-income country setting [5, 32, 46, 49, 59], and one of them was in a lower-middle-income country setting [53]. Studies were published between 2013 and 2021, with sample sizes ranging from 71 to 449. Five studies were conducted in Ethiopia, and three studies had suitable quality study designs. There was no significant association between the duration of ART of more than 5 years compared to ≤5 years (OR = 2.12, 95%CI = 0.95, 4.72) (Fig. 10).

##### The association between time since diagnosis of 5 years or more and disclosure of HIV/AIDS status to infected children

**Fig. 11:**
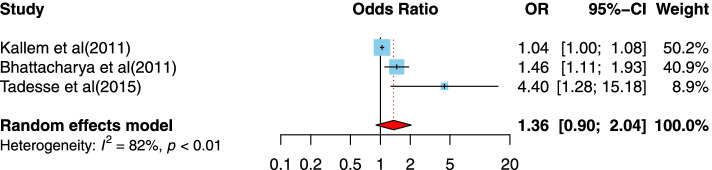
Forest plots for pooled odds ratio of the association between time since diagnosis of 5 years or more compared to less than 5 years and disclosure of HIV/AIDS status to infected children (OR larger than 1 indicates that the odds of being inform on the HIV/AIDS status is higher in children who had time since diagnosis of 5 years or more compared to less than 5 years)

Three cross-sectional studies [47, 53, 62] examined the relationship between time since diagnosis of 5 years or more compared to less than 5 years and disclosure of HIV/AIDS status to infected children. Two studies were conducted in a lower-middle-income country setting [47, 53], and one of them was in a low-income country setting [62]. Studies have been published between 2011 and 2015, with sample sizes ranging from 71 to 177. One of the studies was conducted in Ethiopia, one of them in India, and the other one in Ghana. Two studies had suitable quality designs. As illustrated in Fig. 11, there was no significant association between time since diagnosis of 5 years or more compared to less than 5 years and disclosure of HIV/AIDS status to infected children (OR = 1.36, 95%CI = 0.9, 2.04).

### Publication bias

**Fig. 12:**
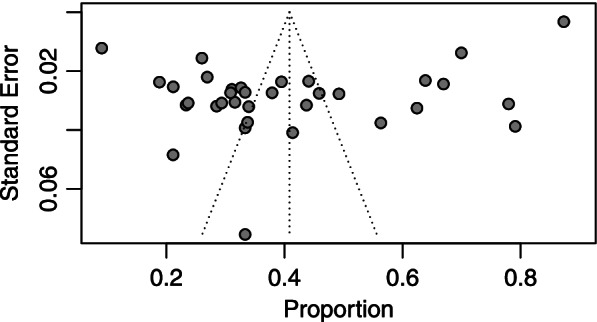
Funnel plot for assessment of publication bias

To identify the possible publication bias, the Egger’s test and the graph were performed. The publication bias test indicates considerable bias based on Eggers test (coefficient = 3.21, *P*-value < 0.001) **(**Fig. 12**)**. Therefore, a met-trim analysis was performed in order to remove the effect of publication bias on the pooled OR. The meta-trim analysis indicated that the pooled OR was 0.11 (95% CI, 0.10–0.22) in the random effect model.

## Discussion

The present systematic review and meta-analysis study aimed to determine the prevalence, socio-demographics and service use determinants associated with the disclosure of HIV/AIDS status to infected children. The current research data identified the older child’s age, primary or lower schooling level, having non-biological parents, social support, the primary educational level of caregivers, ART adherence, and hospital follow-up were significant variables associated with the disclosure of HIV/AIDS status to infected children.

Our results showed that 41% (95% CI: 29–53%) of the HIV positive children in the studies were aware of their positive serostatus. This finding was consistent with those of studies performed in Ethiopia (39.5%) [34] as well as India (41.4%) [47]. Such data consistency might be attributed to the demographically homogeneous samples and the cultural characteristics in both investigations. The age range of the study participants was similar between the study conducted in India and this research. However, the prevalence data for this study were higher than those reported by previous studies in Bahir Dar, Addis Ababa, Kenya, Tanzania, Nigeria, and South Africa, which ranged from 11 to 34% [60, 66–70]. The results of Bahir Dar (31.5%) and Addis Ababa (17.4%) can be explained by variations in the awareness levels of their participants and the advantages that disclosure of HIV status brings. Most caregivers examined in this research were only educated at the primary school level. Contrary to the current study data (i.e., based on the caregivers’ reports alone), the Kenya-based study explored HIV-positive status disclosure based on the reports of the caregivers and children. Accordingly, these differences in measures could have impacted the prevalence rates. The study from South Africa evaluated 4–17-year-olds and families might have believed that the age of children was important for comprehending and understanding the impact of living with HIV. Other studies might have had differences in sociocultural and health services conditions. However, these variations were less than the studies from in North America, Canada, Uganda, and Rwanda where prevalence rates ranged from 10 to 75% [50, 71, 72]. Such data could be explained by differences in healthcare access, favorable parent-child interactions, and sociocultural conditions, leading to improved disclosure status. The findings of the Uganda-based study included older children (age range: 5–18 years) which the increased odds of HIV disclosure in this study can be explained as they were perhaps more mature and keen to know their health status and care givers felt they were more able to deal with the HIV diagnosis. In Rwanda, the difference in the study setting might justify the findings. Patients may have negative feelings about withholding the information, which may affect the therapeutic relationship [73, 74]. Disclosing patient safety incidents may show respect, engage patients in the clinical decision-making process, and improve clinical care [75]. Therefore, effective communication and suitable provision of care after a patient safety incident are necessary to influence patient decisions to initiate legal action [76].

Regarding socio-demographic determinants, in line with previous studies from Gondar (Ethiopia), South Africa, Nigeria, and Uganda [70, 72, 77] we found that children who were aged above 10 years were three times more likely to know their HIV status compared to the children aged 10 years and below. Caregivers in these studies may have considered these children mature enough to understand the disease and possibly had fewer concerns about the disclosure of ‘family secrets’ to others by the child including in school based settings. Our study found the odds of disclosure were greater in children of primary school level or below which is consistent with previous research from a north Indian hospital [47]. We suggest that guidelines for disclosure be developed for caregivers and school authorities that help to prevent discrimination of HIV positive children. The data from our study indicate a non-biological caregivers were more likely to disclose a child’s HIV status than biological parents. The elevated representation of biological parents can be justified by facilitated access to a primary treatment center as well as enhanced adherence time of adults on ART [78]. These results were in line with those of previous investigations suggesting a higher tendency to disclosure by non-biological caregivers [79–81]. There is also literature suggesting that disclosure of HIV status is specifically more challenging for HIV-positive parents who are likely responsible for their children’s infection [79, 80]. The probability of the disclosure of HIV to infected children was higher for children receiving social support from any source compared to their counterparts without such supports [12].

There existed a significant correlation between the caregivers’ level of education and the disclosure of HIV status to children. Consistent with those of research in India [82] illiterate caregivers were two times less likely to disclose the HIV positive status to their children compared to caregivers with at least primary school level of education. It may be that increased levels of education are associated with increased HIV knowledge and awareness of the advantages of revealing the HIV status to their children.

Concerning service use determinants, it was not surprising that children who has been told of their HIV positive status were receiving ART 2.59 times more than those who had not had their status disclosed. This result was in agreement with those of the investigations in Bahir Dar and Ghana [32, 60] and may be associated with to regular visits to the clinic where the children receive their ART. This group was in frequent contact with healthcare professionals and regular counseling services which may have helped to facilitate disclosure of their HIV status. Additionally, over time, children who are taking their ART do not generate symptoms which may lead to declined adherence to pharmacotherapy. As a result, their caregivers might eventually have to disclose their HIV positive status to ensure continued ART adherence. Another determining characteristic affecting disclosure was the offsetting for follow-up. Children who received follow-up at a hospital were 2.8 times more likely to know of their HIV status, compared to their peers who attended community based healthcare centers for follow-up. In line with this observation, another study in Addis Ababa highlighted that the odds of being disclosed were higher among children who were referred from hospitals compared to those visiting healthcare centers [69]. The presence of more knowledgeable and qualified healthcare staff who facilitate disclosure by counseling in hospitals might explain this finding.

### Limitations of the study

There were four key limitations to this meta-analysis. Firstly, most of the included studies were cross-sectional, which may restrict causal and temporal deductions of the relationship between HIV/AIDS status disclosure and other risk factors. These meta-analyses may enhance the statistical inference of analyses and are discussed as reliable sources of evidence. Secondly, most of the studies are from low-income countries in Africa, and no studies from high- and middle-income countries were found and included. Thirdly, few studies investigated the association between independent and dependent variables, emphasizing an important gap in the literature. Finally, we did not interfere with the setting of independent and dependent variables, and so could only report data that were included in the published articles.

### Recommendation for practice and research

In this study, roughly two-third of children (60%) were not aware of their HIV-positive status. Disclosure of HIV to children may vary in different cultures, and is dependent upon the available resources and caregivers’ beliefs as well as available guidelines, protocols and HIV treatments [83]. Many HIV-positive children in this study who were approaching the age of sexual initiation did not have enough knowledge on their status to protect their sex partners. This may mean unintentional transmission of HIV to others and may make promoting prevention strategies difficult [84]. Additionally, several country specific HIV laws [85, 86], require people living with HIV to inform all their sex partners of their status. The utility of HIV criminalization laws as part of HIV prevention is still controversial [87].

In pediatric HIV programs both caregivers and infected children need to be provided with the skills to optimize the benefits of disclosure. Also, interventions that promote HIV disclosure should be culturally appropriate, person centred and mindful of the cognitive and developmental stage of the child. Collaborations between healthcare systems, caregivers, HIV-infected children, and healthcare providers is necessary to improve disclosure adherence that will ultimately achieve better health outcomes for children and adolescents living with HIV.

## Conclusion

The results from this meta-analysis highlight that a significant proportion of children approach adolescence without realizing their HIV positive status. Such data are of importance for HIV health care professionals working in pediatrics. Facilitating HIV diagnosis and disclosure to the infected children and ensuring access to HIV treatment will likely prevent secondary HIV transmission. Healthcare professionals are expected to provide age-appropriate counseling services to this population. They are recommended to present social support and services regarding the disclosure of HIV status to children in accordance with the caregivers’ level of education.

Furthermore, the inequalities are considerable as children are nearly 40% less likely to be on life-saving treatment than adults. Although 5% of all people living with HIV are children, children consist for 15% of all AIDS-related deaths [88]. These findings prove that without attention towards the United Nations Sustainable Development Goal 10 to decrease inequality, progress towards ensuring well-being for all age, especially children, will be hindered for HIV in low and lower-middle countries.

Finally, the findings of the present study may assist to identify the risk groups for low HIV-related knowledge and improve the evidence-based and prevention programmes targeting of HIV education, and also emphasizing on inequalities as barriers to accessing and acquiring HIV prevention information may help to conduct more studies in the future.

## Supplementary Information

**Additional file 1: Supplementary file 1*.*** Search strategy, systematic review and meta-analysis on the disclosure of HIV/AIDS status to infected children, 1985–2021

**Additional file 2: Supplementary file 2.** Risk of bias assessment using Newcastle-Ottawa scale of the studies included in systematic review and meta-analysis on the disclosure of HIV/AIDS status to infected children, 1985–2021

## Data Availability

The datasets used and/or analyzed during the current study are available from the corresponding author on reasonable request.

## Abbreviations

HIV: Human immunodeficiency virus
AIDS: Acquired immune deficiency syndrome
ART: antiretroviral treatment
PRISMA: Protocols of Systematic Reviews and Meta-Analyses
PICO: population, intervention, comparison, outcome
CI: Confidence intervals
NOS: Newcastle-Ottawa Scale
OR: Odds ratio
WHO: World Health Organization

## References

[1] Pinzón-Iregui MC, Beck-Sagué CM (2013). Disclosure of their HIV status to infected children: a review of the literature. J Trop Pediatr.

[2] Xu J-F, Ming Z-Q (2017). Family support, discrimination, and quality of life among ART-treated HIV-infected patients: a two-year study in China. Infect Dis Poverty.

[3] Biraguma J, Mutimura E (2018). Health-related quality of life and associated factors in adults living with HIV in Rwanda. SAHARA J.

[4] Santamaria EK, Dolezal C (2011). Psychosocial implications of HIV serostatus disclosure to youth with perinatally acquired HIV. AIDS patient care and STD.

[5] Abegaz BF, Walle TA (2019). HIV positive status disclosure and associated factor among HIV infected children in pediatric ART clinics in Gondar town public health facilities, North West Ethiopia, 2018. J Infect Public Health.

[6] Madiba S, Diko C (2020). The Consequences of Delaying Telling Children with Perinatal HIV About Their Diagnosis as Perceived by Healthcare Workers in the Eastern Cape; A Qualitative Study. Children.

[7] Vyavaharkar M, Moneyham L (2011). HIV-Disclosure, Social Support, and Depression Among HIV-Infected African American Women Living in the Rural Southeastern United States. AIDS Educ Prev.

[8] Kidia KK, Mupambireyi Z (2014). HIV Status Disclosure to Perinatally-Infected Adolescents in Zimbabwe: A Qualitative Study of Adolescent and Healthcare Worker Perspectives. PLoS One.

[9] Beima-Sofie KM, Brandt L (2017). Pediatric HIV Disclosure Intervention Improves Knowledge and Clinical Outcomes in HIV-Infected Children in Namibia. J Acquir Immune Defic Syndr.

[10] Mutumba M, Musiime V (2015). Disclosure of HIV Status to Perinatally Infected Adolescents in Urban Uganda: A Qualitative Study on Timing, Process, and Outcomes. J Assoc Nurses AIDS Care.

[11] Dahourou D, Raynaud J-P (2018). The challenges of timely and safe HIV disclosure among perinatally HIV-infected adolescents in sub-Saharan Africa. Curr Opin HIV AIDS.

[12] Vreeman RC, Gramelspacher AM (2013). Disclosure of HIV status to children in resource-limited settings: a systematic review. J Int AIDS Soc.

[13] Britto C, Mehta K (2016). Prevalence and Correlates of HIV Disclosure Among Children and Adolescents in Low- and Middle-Income Countries: A Systematic Review. J Dev Behav Pediatr.

[14] Aderomilehin O, Hanciles-Amu A (2016). Perspectives and Practice of HIV Disclosure to Children and Adolescents by Health-Care Providers and Caregivers in sub-Saharan Africa: A Systematic Review. Front Public Health.

[15] Krauss BJ, Letteney S (2016). Why Tell Children: A Synthesis of the Global Literature on Reasons for Disclosing or Not Disclosing an HIV Diagnosis to Children 12 and under. Front Public Health.

[16] Odiachi A (2017). The Impact of Disclosure on Health and Related Outcomes in Human Immunodeficiency Virus-Infected Children: A Literature Review. Front Public Health.

[17] Doat AR, Negarandeh R (2019). Disclosure of HIV Status to Children in Sub-Saharan Africa: A Systematic Review. Medicina (Kaunas).

[18] Dessie G, Wagnew F (2019). The effect of disclosure on adherence to antiretroviral therapy among adults living with HIV in Ethiopia: a systematic review and meta-analysis. BMC Infect Dis.

[19] Smith R, Rossetto K (2008). A meta-analysis of disclosure of one's HIV-positive status, stigma and social support. AIDS Care.

[20] Shamseer L, Moher D (2015). Preferred reporting items for systematic review and meta-analysis protocols (PRISMA-P) 2015: elaboration and explanation. Bmj.

[21] Stroup DF, Berlin JA (2000). Meta-analysis of observational studies in epidemiology: a proposal for reporting. JAMA.

[22] Ghiasvand H, Waye KM (2019). Clinical determinants associated with quality of life for people who live with HIV/AIDS: a Meta-analysis. BMC Health Serv Res.

[23] Ghiasvand H, Bayani A (2018). Comparing injecting and sexual risk behaviors of long-term injectors with new injectors: A meta-analysis. J Addict Dis.

[24] Rezaei O, Ghiasvand H (2020). Factors associated with injecting-related risk behaviors among people who inject drugs: a systematic review and meta-analysis study. J Addict Dis.

[25] Bayat A-H, Mohammadi R (2020). HIV and drug related stigma and risk-taking behaviors among people who inject drugs: a systematic review and meta-analysis. J Addict Dis.

[26] Stang A (2010). Critical evaluation of the Newcastle-Ottawa scale for the assessment of the quality of nonrandomized studies in meta-analyses. Eur J Epidemiol.

[27] Higgins JP, Green S. Cochrane handbook for systematic reviews of interventions, 2nd Edition. Chichester (UK): Wiley; 2019.

[28] Landis JR, Koch GG (1977). The measurement of observer agreement for categorical data. Biometrics.

[29] Begg CB, Mazumdar M (1994). Operating characteristics of a rank correlation test for publication bias. Biometrics.

[30] Egger M, Smith GD (1997). Bias in meta-analysis detected by a simple, graphical test. BMJ.

[31] Appiah SCY, Ivanova O (2021). Disclosure of HIV/AIDS status to infected children in Ghana – A north-south comparison of barriers and enablers. Child Youth Serv Rev.

[32] Alemu A, Berhanu B (2013). Challenges of caregivers to disclose their children’s HIV positive status receiving highly active antiretroviral therapy at pediatric antiretroviral therapy clinics in Bahir Dar, North West Ethiopia. J AIDS Clin Res.

[33] Lencha B, Ameya G (2018). Human immunodeficiency virus infection disclosure status to infected school aged children and associated factors in bale zone, Southeast Ethiopia: cross sectional study. BMC Pediatr.

[34] Negese D, Addis K (2012). HIV-Positive Status Disclosure and Associated Factors among Children in North Gondar, Northwest Ethiopia. ISRN AIDS.

[35] Meena R, Hemal A (2018). Pediatric HIV Disclosure in Northern India: Evaluation of Its Prevalence, Perceptions amongst Caregivers, and Its Impact on CLHIV. AIDS Res Treat.

[36] Bajaria S, Exavery A (2020). Factors Associated with HIV Status Disclosure to Orphans and Vulnerable Children Living with HIV: Results from a Longitudinal Study in Tanzania. AIDS Res Treat.

[37] Bulali RE, Kibusi SM (2018). Factors Associated with HIV Status Disclosure and Its Effect on Treatment Adherence and Quality of Life among Children 6-17 Years on Antiretroviral Therapy in Southern Highlands Zone, Tanzania: Unmatched Case Control Study. Int J Pediatr.

[38] Namasopo-Oleja MS, Bagenda D (2015). Factors affecting disclosure of serostatus to children attending Jinja Hospital Paediatric HIV clinic, Uganda. Afr Health Sci.

[39] Atwiine B, Kiwanuka J (2015). Understanding the role of age in HIV disclosure rates and patterns for HIV-infected children in southwestern Uganda. AIDS Care.

[40] Mengesha MM, Dessie Y (2018). Perinatally acquired HIV-positive status disclosure and associated factors in Dire Dawa and Harar, Eastern Ethiopia: a health facility-based cross-sectional study. BMJ Open.

[41] Vreeman RC, Scanlon ML (2015). Characteristics of HIV-infected adolescents enrolled in a disclosure intervention trial in western Kenya. AIDS Care.

[42] Paintsil E, Kyriakides TC (2020). Clinic-Based Pediatric Disclosure Intervention Trial Improves Pediatric HIV Status Disclosure in Ghana. J Acquir Immune Defic Syndr.

[43] Cluver LD, Hodes RJ (2015). 'HIV is like a tsotsi. ARVs are your guns': associations between HIV-disclosure and adherence to antiretroviral treatment among adolescents in South Africa. Aids.

[44] Nzota MS, Matovu JK (2015). Determinants and processes of HIV status disclosure to HIV--infected children aged 4 to 17 years receiving HIV care services at Baylor College of Medicine Children's Foundation Tanzania, Centre of Excellence (COE) in Mbeya: a cross-sectional study. BMC Pediatr.

[45] Ayele WM (2021). Determinants of HIV/AIDS Disclosure in Pediatrics Age from 5-14 Years on ART. J AIDS Clin Res.

[46] Tamir Y, Aychiluhem M (2014). Disclosure status and associated factors among children living with HIV in east Gojjam, northwest of Ethiopia. Qual Prim Care.

[47] Bhattacharya M, Dubey AP (2011). Patterns of diagnosis disclosure and its correlates in HIV-Infected North Indian children. J Trop Pediatr.

[48] Okechukwu AA (2018). OUaEE: Disclosure of HIV Status to Infected Children and Adolescents by Their Parents/Caregivers in a Tertiary Health Facility in Abuja, Nigeria. Austin J HIV/AIDS Res.

[49] Guta A, Areri HA (2020). HIV-positive status disclosure and associated factors among children in public health facilities in Dire Dawa, Eastern Ethiopia: A cross-sectional study. PLoS One.

[50] Madiba S, Mahloko J (2013). Prevalence and factors associated with disclosure of HIV diagnosis to infected children receiving antiretroviral treatment in public health care facilities in Gauteng, South Africa. J Clin Res HIV AIDS Prev.

[51] Shallo SA, Tassew M (2020). HIV Positive Status Disclosure and Its Associated Factors Among Children on Antiretroviral Therapy in West Shoa Zone, Western Ethiopia, 2019: A Mixed Method Cross-Sectional Study. J Multidiscip Healthc.

[52] Finnegan A, Langhaug L (2019). The prevalence and process of pediatric HIV disclosure: A population-based prospective cohort study in Zimbabwe. PLoS One.

[53] Kallem S, Renner L (2011). Prevalence and pattern of disclosure of HIV status in HIV-infected children in Ghana. AIDS Behav.

[54] Madiba S, Mokgatle M (2017). Fear of stigma, beliefs, and knowledge about HIV are barriers to early access to HIV testing and disclosure for perinatally infected children and adolescents in rural communities in South Africa. S Afr Fam Pract.

[55] Danjuma JS, Sambo MN (2020). Predictors of pediatric HIV disclosure among caregivers of HIV positive children attending special treatment clinic in dalhatu araf specialist hospital, Lafia, Nigeria. Niger J Clin Pract.

[56] John-Stewart GC, Wariua G (2013). Prevalence, perceptions, and correlates of pediatric HIV disclosure in an HIV treatment program in Kenya. AIDS Care.

[57] Murnane PM, Sigamoney SL (2017). Extent of disclosure: what perinatally HIV-infected children have been told about their own HIV status. AIDS Care.

[58] Odiachi A, Abegunde D (2016). Prevalence and predictors of pediatric disclosure among HIV-infected Nigerian children on treatment. AIDS Care.

[59] Tucho WA, Tekelehaimanot AN (2021). Disclosure Status and Associated Factors Among Children on Antiretroviral Therapy in Ethiopia. Pediatric Health Med Ther.

[60] Biadgilign S, Deribew A (2011). Factors associated with HIV/AIDS diagnostic disclosure to HIV infected children receiving HAART: a multi-center study in Addis Ababa, Ethiopia. PLoS One.

[61] Vreeman RC, Scanlon ML (2014). A cross-sectional study of disclosure of HIV status to children and adolescents in western Kenya. PLoS One.

[62] Tadesse BT, Foster BA (2015). Cross Sectional Characterization of Factors Associated with Pediatric HIV Status Disclosure in Southern Ethiopia. PLoS One.

[63] Kalembo FW, Kendall GE (2019). Socio-demographic, clinical, and psychosocial factors associated with primary caregivers' decisions regarding HIV disclosure to their child aged between 6 and 12 years living with HIV in Malawi. PLoS One.

[64] Sirikum C, Sophonphan J (2014). HIV disclosure and its effect on treatment outcomes in perinatal HIV-infected Thai children. AIDS Care.

[65] van Elsland SL, Peters RPH (2019). Disclosure of human immunodeficiency virus status to children in South Africa: A comprehensive analysis. South Afr J HIV Med.

[66] Atwiine B, Kiwanuka J (2015). Understanding the role of age in HIV disclosure rates and patterns for HIV-infected children in southwestern Uganda. AIDS Care.

[67] Namasopo-Oleja SM, Bagenda D (2015). Factors affecting disclosure of serostatus to children attending Jinja Hospital Paediatric HIV clinic, Uganda. Afr Health Sci.

[68] Kallem S, Renner L (2011). Prevalence and pattern of disclosure of HIV status in HIV-infected children in Ghana. AIDS Behav.

[69] Turissini ML, Nyandiko WM (2013). The prevalence of disclosure of HIV status to HIV-infected children in Western Kenya. J Pediatric Infect Dis Soc.

[70] Negese D, Addis K (2012). HIV-positive status disclosure and associated factors among children in North Gondar, Northwest Ethiopia. ISRN AIDS.

[71] Wilfert C, Beck D (1999). Disclosure of illness status to children and adolescents with HIV infection. Pediatrics.

[72] John-Stewart GC, Wariua G (2013). Prevalence, perceptions, and correlates of pediatric HIV disclosure in an HIV treatment program in Kenya. AIDS Care.

[73] Gallagher TH, Levinson W (2005). Disclosing harmful medical errors to patients: a time for professional action. Arch Intern Med.

[74] Witman AB, Park DM (1996). How do patients want physicians to handle mistakes?: A survey of internal medicine patients in an academic setting. Arch Intern Med.

[75] Management ASfHR (2004). Disclosure: what works now and what can work even better. J Healthc Risk Manag.

[76] Wu AW, Boyle DJ (2013). Disclosure of adverse events in the United States and Canada: an update, and a proposed framework for improvement. J Public Health Res.

[77] Mumburi LP, Hamel BC (2014). Factors associated with HIV-status disclosure to HIV-infected children receiving care at Kilimanjaro Christian Medical Centre in Moshi, Tanzania. Pan Afr Med J.

[78] Myer L, Moodley K (2006). Healthcare providers' perspectives on discussing HIV status with infected children. J Trop Pediatr.

[79] Mellins CA, Brackis-Cott E (2002). Patterns of HIV status disclosure to perinatally HIV-infected children and subsequent mental health outcomes. Clin Child Psychol Psychiatry.

[80] Thorne C, Newell M-L (2002). Older children and adolescents surviving with vertically acquired HIV infection. J Acquir Immune Defic Syndr.

[81] Lesch A, Swartz L (2007). Paediatric HIV/AIDS disclosure: towards a developmental and process-oriented approach. AIDS Care.

[82] Oberdorfer P, Puthanakit T (2006). Disclosure of HIV/AIDS diagnosis to HIV-infected children in Thailand. J Paediatr Child Health.

[83] Bulali RE, Kibusi SM (2018). Factors associated with hiv status disclosure and its effect on treatment adherence and quality of life among children 6–17 years on antiretroviral therapy in southern highlands zone, Tanzania: unmatched case control study. Int J Pediatr.

[84] Lencha B, Ameya G (2018). Human immunodeficiency virus infection disclosure status to infected school aged children and associated factors in bale zone, Southeast Ethiopia: cross sectional study. BMC Pediatr.

[85] Galletly CL, DiFranceisco W (2009). HIV-positive persons’ awareness and understanding of their state’s criminal HIV disclosure law. AIDS Behav.

[86] Bernard E (2005). Prosecutions for HIV exposure and transmission on the rise throughout Europe.

[87] Chenneville T, Lynn V (2015). Disclosure of HIV status among female youth with HIV. Ethics Behav.

[88] New report reveals stark inequalities in access to HIV prevention and treatment services for children—partners call for urgent action. https://www.who.int/news/item/21-07-2021-new-report-reveals-stark-inequalities-in-access-to-hiv-prevention-and-treatment-services-for-children-partners-call-for-urgent-action. Accessed 21 July 2021.

