# Supplementary material for: Prevalence, socio-demographics and service use determinants associated with disclosure of HIV/AIDS status to infected children: a systematic review and meta-analysis by 1985–2021
Source: Arch Public Health. 2022 Jun 9;80:154. doi: 10.1186/s13690-022-00910-6 (PMC9178876; doi:10.1186/s13690-022-00910-6)
Supplement: Supplementary file 1 — Additional file 1: Supplementary file 1. Search strategy, systematic review and meta-analysis on the disclosure of HIV/AIDS status to infected children, 1985–2021 [file 13690_2022_910_MOESM1_ESM.docx]

***Supplementary file 1. Search strategy, systematic review and meta-analysis on the disclosure of HIV/AIDS status to infected children, 1985-2021***

| *PubMed Search* | | |
| --- | --- | --- |
| *Search number* | *Query* | *Item founds* |
| *29* | ((((((((((((((((((((Social Determinants of Health[MeSH Terms]) OR (Socioeconomic Factors[MeSH Terms])) OR (Spouses[MeSH Terms])) OR (Literacy[MeSH Terms])) OR (Medication Adherence[MeSH Terms])) OR (CD4 Lymphocyte Count[MeSH Terms])) OR (Health Services Accessibility[MeSH Terms])) OR (Time-to-Treatment[MeSH Terms])) OR (Time to diagnosis[MeSH Terms])) OR (Time to diagnosis[Title/Abstract])) OR (previous testing[Title/Abstract])) OR (previous testing[Title/Abstract])) OR (Social Stigma[MeSH Terms])) OR (Stigma[MeSH Terms])) OR (Self-Disclosure[MeSH Terms])) OR (Self-Concept[MeSH Terms])) OR (Anti-HIV Agents[MeSH Terms])) OR (Antiretroviral Therapy, Highly Active[MeSH Terms])) OR (Social Support[MeSH Terms])) AND (((people who lived with HIV[Title/Abstract]) OR (living with hiv[Title/Abstract])) OR (HIV[MeSH Terms]))) AND ((((Child Day Care Centers[MeSH Terms]) OR (Child[MeSH Terms])) OR (Adult Children[MeSH Terms])) OR (Child, Preschool[MeSH Terms])) |  |
| *28* | (((Child Day Care Centers[MeSH Terms]) OR (Child[MeSH Terms])) OR (Adult Children[MeSH Terms])) OR (Child, Preschool[MeSH Terms]) |  |
| *27* | ((people who lived with HIV[Title/Abstract]) OR (living with hiv[Title/Abstract])) OR (HIV[MeSH Terms]) |  |
| 26 | ((((((((((((((((((Social Determinants of Health[MeSH Terms]) OR (Socioeconomic Factors[MeSH Terms])) OR (Spouses[MeSH Terms])) OR (Literacy[MeSH Terms])) OR (Medication Adherence[MeSH Terms])) OR (CD4 Lymphocyte Count[MeSH Terms])) OR (Health Services Accessibility[MeSH Terms])) OR (Time-to-Treatment[MeSH Terms])) OR (Time to diagnosis[MeSH Terms])) OR (Time to diagnosis[Title/Abstract])) OR (previous testing[Title/Abstract])) OR (previous testing[Title/Abstract])) OR (Social Stigma[MeSH Terms])) OR (Stigma[MeSH Terms])) OR (Self-Disclosure[MeSH Terms])) OR (Self-Concept[MeSH Terms])) OR (Anti-HIV Agents[MeSH Terms])) OR (Antiretroviral Therapy, Highly Active[MeSH Terms])) OR (Social Support[MeSH Terms]) |  |
| 25 | Social Support[MeSH Terms] |  |
| 24 | Antiretroviral Therapy, Highly Active[MeSH Terms] |  |
| 23 | Anti-HIV Agents[MeSH Terms] |  |
| 22 | HIV[MeSH Terms] |  |
| 21 | Child, Preschool[MeSH Terms] |  |
| 20 | Adult Children[MeSH Terms] |  |
| 19 | Child[MeSH Terms] |  |
| 18 | Child Day Care Centers[MeSH Terms] |  |
| 17 | living with hiv[Title/Abstract] |  |
| 16 | people who lived with HIV[Title/Abstract] |  |
| 15 | Self-Concept[MeSH Terms] |  |
| 14 | Self-Disclosure[MeSH Terms] |  |
| 13 | Stigma[MeSH Terms] |  |
| 12 | Social Stigma[MeSH Terms] |  |
| 11 | previous testing[Title/Abstract] |  |
| 10 | Time to diagnosis[Title/Abstract] |  |
| 9 | Time to diagnosis[MeSH Terms] |  |
| 8 | Time-to-Treatment[MeSH Terms] |  |
| 7 | Health Services Accessibility[MeSH Terms] |  |
| 6 | CD4 Lymphocyte Count[MeSH Terms] |  |
| 5 | Medication Adherence[MeSH Terms] |  |
| 4 | Literacy[MeSH Terms] |  |
| 3 | Spouses[MeSH Terms] |  |
| 2 | Socioeconomic Factors[MeSH Terms] |  |
| 1 | Social Determinants of Health[MeSH Terms] |  |
| Cochrane search | |  |
| *ID* | *Search* |  |
| #1 | MeSH descriptor: [HIV] explode all trees |  |
| #2 | (people who lived with HIV):ti (Word variations have been searched) |  |
| #3 | (Living with HIV):ti (Word variations have been searched) |  |
| #4 | (stigma):ti (Word variations have been searched) |  |
| #5 | MeSH descriptor: [Social Stigma] explode all trees |  |
| #6 | MeSH descriptor: [Shame] explode all trees |  |
| #7 | MeSH descriptor: [Self Disclosure] explode all trees |  |
| #8 | MeSH descriptor: [Self Concept] explode all trees |  |
| #9 | (Negative Self-Image):ti (Word variations have been searched) |  |
| #10 | MeSH descriptor: [Antiretroviral Therapy, Highly Active] explode all trees |  |
| #11 | (feel guilty):ti (Word variations have been searched) |  |
| #12 | MeSH descriptor: [Social Determinants of Health] explode all trees |  |
| #13 | MeSH descriptor: [Socioeconomic Factors] explode all trees |  |
| #14 | MeSH descriptor: [Spouses] explode all trees |  |
| #15 | MeSH descriptor: [Literacy] explode all trees |  |
| #16 | MeSH descriptor: [Medication Adherence] explode all trees |  |
| #17 | MeSH descriptor: [CD4 Lymphocyte Count] explode all trees |  |
| #18 | MeSH descriptor: [Health Services Accessibility] explode all trees |  |
| #19 | MeSH descriptor: [Time-to-Treatment] explode all trees |  |
| #20 | (Time to diagnosis):ti (Word variations have been searched) |  |
| #21 | (previous testing):ti (Word variations have been searched) |  |
| #22 | MeSH descriptor: [Anti-HIV Agents] explode all trees |  |
| #23 | MeSH descriptor: [Child, Preschool] explode all trees |  |
| #24 | MeSH descriptor: [Adult Children] explode all trees |  |
| #25 | MeSH descriptor: [Child] explode all trees |  |
| #26 | MeSH descriptor: [Child Day Care Centers] explode all trees |  |
| #27 | #1 OR #2 OR #3 |  |
| #28 | #4 OR #5 OR #5 OR #6 OR #7 OR #8 OR #9 OR #10 OR #11 OR #12 OR #13 OR #14 OR #15 OR #16 OR #17 OR #18 OR #19 OR #20 OR #21 OR #22 |  |
| #29 | #23 OR #24 OR #25 OR #26 |  |
| #30 | #27 AND #28 AND #29 |  |
| *Web of knowledge* | |  |
| *#29* | *#26  AND #27 AND #28*  *Indexes=SCI-EXPANDED, SSCI, A&HCI, CPCI-S, CPCI-SSH, BKCI-S, BKCI-SSH, ESCI, CCR-EXPANDED, IC Timespan=All years* |  |
| *#28* | *#22 OR #23 OR #24 OR #25*  *Indexes=SCI-EXPANDED, SSCI, A&HCI, CPCI-S, CPCI-SSH, BKCI-S, BKCI-SSH, ESCI, CCR-EXPANDED, IC Timespan=All years* |  |
| *#27* | *#19 OR #20 OR #21*  *Indexes=SCI-EXPANDED, SSCI, A&HCI, CPCI-S, CPCI-SSH, BKCI-S, BKCI-SSH, ESCI, CCR-EXPANDED, IC Timespan=All years* |  |
| *#26* | *#1 OR #2 OR #3 OR #4 OR #5 OR #6 OR #7 OR #8 OR #9 OR #10 #11 OR #12 OR #13 OR #14 OR #15 OR #16 OR #17 OR #18*  *Indexes=SCI-EXPANDED, SSCI, A&HCI, CPCI-S, CPCI-SSH, BKCI-S, BKCI-SSH, ESCI, CCR-EXPANDED, IC Timespan=All years* |  |
| *#25* | *TI= (*Child *Day Care Centers)*  *Indexes=SCI-EXPANDED, SSCI, A&HCI, CPCI-S, CPCI-SSH, BKCI-S, BKCI-SSH, ESCI, CCR-EXPANDED, IC Timespan=All years* |  |
| *#24* | *TI=(*Child*)*  *Indexes=SCI-EXPANDED, SSCI, A&HCI, CPCI-S, CPCI-SSH, BKCI-S, BKCI-SSH, ESCI, CCR-EXPANDED, IC Timespan=All years* |  |
| *#23* | *TI= (*Adult *Children)*  *Indexes=SCI-EXPANDED, SSCI, A&HCI, CPCI-S, CPCI-SSH, BKCI-S, BKCI-SSH, ESCI, CCR-EXPANDED, IC Timespan=All years* |  |
| *#22* | *TI=(*Child*, Preschool)*  *Indexes=SCI-EXPANDED, SSCI, A&HCI, CPCI-S, CPCI-SSH, BKCI-S, BKCI-SSH, ESCI, CCR-EXPANDED, IC Timespan=All years* |  |
| *#21* | *TI=(HIV)*  *Indexes=SCI-EXPANDED, SSCI, A&HCI, CPCI-S, CPCI-SSH, BKCI-S, BKCI-SSH, ESCI, CCR-EXPANDED, IC Timespan=All years* |  |
| *#20* | *TI=(Living with  HIV)*  *Indexes=SCI-EXPANDED, SSCI, A&HCI, CPCI-S, CPCI-SSH, BKCI-S, BKCI-SSH, ESCI, CCR-EXPANDED, IC Timespan=All years* |  |
| *#19* | *TI=(people who  lived  with  HIV)*  *Indexes=SCI-EXPANDED, SSCI, A&HCI, CPCI-S, CPCI-SSH, BKCI-S, BKCI-SSH, ESCI, CCR-EXPANDED, IC Timespan=All years* |  |
| *#18* | *TI=(* *Anti-HIV Agents)*  *Indexes=SCI-EXPANDED, SSCI, A&HCI, CPCI-S, CPCI-SSH, BKCI-S, BKCI-SSH, ESCI, CCR-EXPANDED, IC Timespan=All years* |  |
| *#17* | *TI=(feel guilty)*  *Indexes=SCI-EXPANDED, SSCI, A&HCI, CPCI-S, CPCI-SSH, BKCI-S, BKCI-SSH, ESCI, CCR-EXPANDED, IC Timespan=All years* |  |
| *#16* | *TI=(* *Antiretroviral Therapy, Highly Active)*  *Indexes=SCI-EXPANDED, SSCI, A&HCI, CPCI-S, CPCI-SSH, BKCI-S, BKCI-SSH, ESCI, CCR-EXPANDED, IC Timespan=All years* |  |
| *#15* | *TI=( Social Support)*  *Indexes=SCI-EXPANDED, SSCI, A&HCI, CPCI-S, CPCI-SSH, BKCI-S, BKCI-SSH, ESCI, CCR-EXPANDED, IC Timespan=All years* |  |
| *#14* | *TI=(Self Concept)*  *Indexes=SCI-EXPANDED, SSCI, A&HCI, CPCI-S, CPCI-SSH, BKCI-S, BKCI-SSH, ESCI, CCR-EXPANDED, IC Timespan=All years* |  |
| *#13* | *TI=(Self Disclosure)*  *Indexes=SCI-EXPANDED, SSCI, A&HCI, CPCI-S, CPCI-SSH, BKCI-S, BKCI-SSH, ESCI, CCR-EXPANDED, IC Timespan=All years* |  |
| *#12* | *TI=(Stigma)*  *Indexes=SCI-EXPANDED, SSCI, A&HCI, CPCI-S, CPCI-SSH, BKCI-S, BKCI-SSH, ESCI, CCR-EXPANDED, IC Timespan=All years* |  |
| *#11* | *TI=(Social Stigma)*  *Indexes=SCI-EXPANDED, SSCI, A&HCI, CPCI-S, CPCI-SSH, BKCI-S, BKCI-SSH, ESCI, CCR-EXPANDED, IC Timespan=All years* |  |
| *#10* | *TI=( previous testing)*  *Indexes=SCI-EXPANDED, SSCI, A&HCI, CPCI-S, CPCI-SSH, BKCI-S, BKCI-SSH, ESCI, CCR-EXPANDED, IC Timespan=All years* |  |
| *#9* | *TI=(* Time to diagnosis*)*  *Indexes=SCI-EXPANDED, SSCI, A&HCI, CPCI-S, CPCI-SSH, BKCI-S, BKCI-SSH, ESCI, CCR-EXPANDED, IC Timespan=All years* |  |
| *#8* | *TI=( Time-to-Treatment)*  *Indexes=SCI-EXPANDED, SSCI, A&HCI, CPCI-S, CPCI-SSH, BKCI-S, BKCI-SSH, ESCI, CCR-EXPANDED, IC Timespan=All years* |  |
| *#7* | *TI=( Health Services Accessibility)*  *Indexes=SCI-EXPANDED, SSCI, A&HCI, CPCI-S, CPCI-SSH, BKCI-S, BKCI-SSH, ESCI, CCR-EXPANDED, IC Timespan=All years* |  |
| *#6* | *TI=(* *CD4 Lymphocyte Count)*  *Indexes=SCI-EXPANDED, SSCI, A&HCI, CPCI-S, CPCI-SSH, BKCI-S, BKCI-SSH, ESCI, CCR-EXPANDED, IC Timespan=All years* |  |
| *#5* | *TI=(* *Medication Adherence)*  *Indexes=SCI-EXPANDED, SSCI, A&HCI, CPCI-S, CPCI-SSH, BKCI-S, BKCI-SSH, ESCI, CCR-EXPANDED, IC Timespan=All years* |  |
| *#4* | *TI=(* *Literacy)*  *Indexes=SCI-EXPANDED, SSCI, A&HCI, CPCI-S, CPCI-SSH, BKCI-S, BKCI-SSH, ESCI, CCR-EXPANDED, IC Timespan=All years* |  |
| *#3* | *TI=(* *Spouses)*  *Indexes=SCI-EXPANDED, SSCI, A&HCI, CPCI-S, CPCI-SSH, BKCI-S, BKCI-SSH, ESCI, CCR-EXPANDED, IC Timespan=All years* |  |
| *#2* | *TI=(* *Socioeconomic Factors)*  *Indexes=SCI-EXPANDED, SSCI, A&HCI, CPCI-S, CPCI-SSH, BKCI-S, BKCI-SSH, ESCI, CCR-EXPANDED, IC Timespan=All years* |  |
| *#1* | *TI=(* *Social Determinants of Health)*  *Indexes=SCI-EXPANDED, SSCI, A&HCI, CPCI-S, CPCI-SSH, BKCI-S, BKCI-SSH, ESCI, CCR-EXPANDED, IC Timespan=All years* |  |

|  | Scopus search | Item found |
| --- | --- | --- |
|  | ( ( ( TITLE-ABS-KEY ( social AND determinants AND of AND health ) ) OR ( TITLE-ABS-KEY ( socioeconomic AND factors ) ) OR ( TITLE-ABS-KEY ( spouses ) ) OR ( TITLE-ABS-KEY ( literacy ) ) ) OR ( ( TITLE-ABS-KEY ( medication AND adherence ) ) OR ( TITLE-ABS-KEY ( cd4 AND lymphocyte AND count ) ) OR ( TITLE-ABS-KEY ( health AND services AND accessibility ) ) OR ( TITLE-ABS-KEY ( time-to-treatment ) ) OR ( TITLE-ABS-KEY ( time AND to AND diagnosis ) ) OR ( TITLE-ABS-KEY ( previous AND testing ) ) ) ) AND ( TITLE-ABS-KEY ( hiv ) ) AND ( ( TITLE-ABS-KEY ( stigma ) ) OR ( TITLE-ABS-KEY ( social AND stigma ) ) OR ( TITLE-ABS-KEY ( shame ) ) OR ( TITLE-ABS-KEY ( self AND disclosure ) ) OR ( TITLE-ABS-KEY ( self AND concept ) ) OR ( TITLE-ABS-KEY ( negative AND self-image ) ) OR ( TITLE-ABS-KEY ( blame ) ) OR ( TITLE-ABS-KEY ( feel AND guilty ) ) ) AND ( ( TITLE-ABS-KEY ( people AND who AND lived AND with AND hiv ) ) OR ( TITLE-ABS-KEY ( living AND with AND hiv ) ) ) ...View More |  |
|  | ( ( TITLE-ABS-KEY ( social AND determinants AND of AND health ) ) OR ( TITLE-ABS-KEY ( socioeconomic AND factors ) ) OR ( TITLE-ABS-KEY ( spouses ) ) OR ( TITLE-ABS-KEY ( literacy ) ) ) OR ( ( TITLE-ABS-KEY ( medication AND adherence ) ) OR ( TITLE-ABS-KEY ( cd4 AND lymphocyte AND count ) ) OR ( TITLE-ABS-KEY ( health AND services AND accessibility ) ) OR ( TITLE-ABS-KEY ( time-to-treatment ) ) OR ( TITLE-ABS-KEY ( time AND to AND diagnosis ) ) OR ( TITLE-ABS-KEY ( previous AND testing ) ) ) ...View More |  |
|  | ( TITLE-ABS-KEY ( child AND day AND care AND centers ) OR TITLE-ABS-KEY ( child ) OR TITLE-ABS-KEY ( adult AND children ) OR TITLE-ABS-KEY ( child, AND preschool ) ) |  |
|  | ( TITLE-ABS-KEY ( people AND who AND lived AND with AND hiv ) ) OR ( TITLE-ABS-KEY ( living AND with AND hiv ) OR (TITLE-ABS-KEY (HIV)) |  |
|  | ( TITLE-ABS-KEY ( stigma ) ) OR ( TITLE-ABS-KEY ( social AND stigma ) ) OR ( TITLE-ABS-KEY ( shame ) ) OR ( TITLE-ABS-KEY ( self AND disclosure ) ) OR ( TITLE-ABS-KEY ( self AND concept ) ) OR ( TITLE-ABS-KEY ( negative AND self-image ) ) OR ( TITLE-ABS-KEY ( blame ) ) OR ( TITLE-ABS-KEY ( feel AND guilty ) ) |  |
|  | ( TITLE-ABS-KEY ( medication AND adherence ) ) OR ( TITLE-ABS-KEY ( cd4 AND lymphocyte AND count ) ) OR ( TITLE-ABS-KEY ( health AND services AND accessibility ) ) OR ( TITLE-ABS-KEY ( time-to-treatment ) ) OR ( TITLE-ABS-KEY ( time AND to AND diagnosis ) ) OR ( TITLE-ABS-KEY ( previous AND testing ) ) |  |
|  | ( TITLE-ABS-KEY ( social AND determinants AND of AND health ) ) OR ( TITLE-ABS-KEY ( socioeconomic AND factors ) ) OR ( TITLE-ABS-KEY ( spouses ) ) OR ( TITLE-ABS-KEY ( literacy ) ) |  |
|  | TITLE-ABS-KEY ( living AND with AND hiv ) |  |
|  | TITLE-ABS-KEY ( people AND who AND lived AND with AND hiv ) |  |
|  | TITLE-ABS-KEY ( feel AND guilty ) |  |
|  | TITLE-ABS-KEY ( blame ) |  |
|  | TITLE-ABS-KEY ( negative AND self-image ) |  |
|  | TITLE-ABS-KEY ( self AND concept ) |  |
|  | TITLE-ABS-KEY ( self AND disclosure ) |  |
|  | TITLE-ABS-KEY ( shame ) |  |
|  | TITLE-ABS-KEY ( social AND stigma ) |  |
|  | TITLE-ABS-KEY ( hiv ) |  |
|  | TITLE-ABS-KEY ( stigma ) |  |
|  | TITLE-ABS-KEY ( hiv ) |  |
|  | TITLE-ABS-KEY ( previous AND testing ) |  |
|  | TITLE-ABS-KEY ( time AND to AND diagnosis ) |  |
|  | TITLE-ABS-KEY ( time-to-treatment ) |  |
|  | TITLE-ABS-KEY ( health AND services AND accessibility ) |  |
|  | TITLE-ABS-KEY ( cd4 AND lymphocyte AND count ) |  |
|  | TITLE-ABS-KEY ( medication AND adherence ) |  |
|  | TITLE-ABS-KEY ( literacy ) |  |
|  | TITLE-ABS-KEY ( spouses ) |  |
|  | TITLE-ABS-KEY ( socioeconomic AND factors ) |  |
