# Supplementary material for: Prevalence, socio-demographics and service use determinants associated with disclosure of HIV/AIDS status to infected children: a systematic review and meta-analysis by 1985–2021
Source: Arch Public Health. 2022 Jun 9;80:154. doi: 10.1186/s13690-022-00910-6 (PMC9178876; doi:10.1186/s13690-022-00910-6)
Supplement: Supplementary file 2 — Additional file 2: Supplementary file 2. Risk of bias assessment using Newcastle-Ottawa scale of the studies included in systematic review and meta-analysis on the disclosure of HIV/AIDS status to infected children, 1985–2021 [file 13690_2022_910_MOESM2_ESM.docx]

***Supplementary file 2. Risk of bias assessment using Newcastle-Ottawa scale of the studies included in systematic review and meta-analysis on the disclosure of HIV/AIDS status to infected children, 1985-2021***

| **Study** | **Selection**  **(***)** | **Comparability**  **(*)** | **Exposure/outcome**  **(*)** | **Quality Assessment** | **Quality Assessment score** |
| --- | --- | --- | --- | --- | --- |
| Appiah et al[[1](#_ENREF_1)] | *** |  | * | Good | 4 |
| Abegaz et al[[2](#_ENREF_2)] | * | * | * | Satisfactory | 3 |
| Alemu et al[[3](#_ENREF_3)] | ** | * | * | Good | 4 |
| Lencha et al[[4](#_ENREF_4)] | *** |  | * | Good | 4 |
| Negese et al[[5](#_ENREF_5)] | *** | * |  | Good | 4 |
| Meena et al[[6](#_ENREF_6)] | * | * | * | Satisfactory | 3 |
| Bajaria et al[[7](#_ENREF_7)] | * | * | * | Satisfactory | 3 |
| Bulali et al[[8](#_ENREF_8)] | ** | * | * | Good | 4 |
| Namasopo-Oleja et al[[9](#_ENREF_9)] | *** | * | * | Very Good | 5 |
| Atwiine et al[[10](#_ENREF_10)] | ** | * | * | Good | 4 |
| Mengesha et al[[11](#_ENREF_11)] | ** | * | * | Good | 4 |
| Vreeman et al[[12](#_ENREF_12)] | *** | * | * | Very Good | 5 |
| Paintsil et al[[13](#_ENREF_13)] | ** | * | * | Good | 4 |
| Cluver et al[[14](#_ENREF_14)] | ** | * | * | Good | 4 |
| Nzota et al[[15](#_ENREF_15)] | ** | * | * | Good | 4 |
| Ayele et al[[16](#_ENREF_16)] | *** | * | * | Very Good | 5 |
| Tamir et al[[17](#_ENREF_17)] | *** | * | * | Very Good | 5 |
| Bhattacharya et al[[18](#_ENREF_18)] | *** | * | * | Very Good | 5 |
| Okechukwu et al[[19](#_ENREF_19)] | ** | * | * | Good | 4 |
| Guta et al[[20](#_ENREF_20)] | * | * | * | Satisfactory | 3 |
| van Elsland et al[[21](#_ENREF_21)] | ** | * | * | Good | 4 |
| Madiba et al[[22](#_ENREF_22)] | ** | * | * | Good | 4 |
| Shallo and Tassew[[23](#_ENREF_23)] | *** | * | * | Very Good | 5 |
| Finnegan et al[[24](#_ENREF_24)] | *** | * | * | Very Good | 5 |
| Kallem et al[[25](#_ENREF_25)] | ** | * | * | Good | 4 |
| Madiba and Mokgatle[[26](#_ENREF_26)] | ** | * | * | Good | 4 |
| Danjuma et al[[27](#_ENREF_27)] | * | * | * | Satisfactory | 3 |
| John-Stewart et al[[28](#_ENREF_28)] | ** | * | * | Good | 4 |
| Murnane et al[[29](#_ENREF_29)] | ** | * | * | Good | 4 |
| Odiachi and Abegunde[[30](#_ENREF_30)] | ** | * | * | Good | 4 |
| Beima-Sofie et al[[31](#_ENREF_31)] | *** | * | * | Very Good | 5 |
| Tucho et al[[32](#_ENREF_32)] | *** |  | * | Good | 4 |
| Biadgilign et al[[33](#_ENREF_33)] | *** | * | * | Very Good | 4 |
| Vreeman et al[[34](#_ENREF_34)] | *** | * | * | Very Good | 5 |
| Tadesse et al[[35](#_ENREF_35)] | ** | * | * | Good | 4 |
| Kalembo et al[[36](#_ENREF_36)] | ** | * | * | Good | 4 |
| Sirikum et al[[37](#_ENREF_37)] | *** |  | * | Good | 4 |
